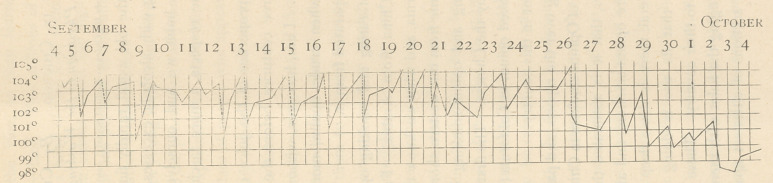# Antipyrin in Typhoid Fever

**Published:** 1886-02

**Authors:** M. Mannheimer

**Affiliations:** Chicago, Physician to Michael Reese and Alexian Brothers’ Hospitals, Chicago


### THE CHICAGO MEDICAL JOURNAL AND EXAMINER.

VOL. LII. FEBRUARY, 1886. NO. 2.

### ORIGINAL COMMUNICATIONS.

### CLINICAL OBSERVATIONS ON THE EMPLOYMENT OF ANTIPYRIN IN TYPHOID FEVER.

DR. M. MANNHEIMER, *of Chicago, Physician to Michael Reese and Alexian Brothers' Hospitals, Chicago.*

Encouraged by accounts which have appeared in European journals, relative to the usefulness of antipyrin when exhibited in typhoid fever, I lately introduced the remedy in the practice of the Alexian Brothers' and the Michael Reese Hospitals in this city.

With the assistance of Doctors Clevenger, Hoelcher, Frankenthal and Collins, 175 cases of typhoid fever were treated in these institutions during the year 1885, the resulting death rate being 1.71 per cent.

One hundred and thirty-nine of these patients were cared for at the Alexian Hospital, of whom three died; and thirty-six patients entered and were discharged convalescent from the Michael Reese Hospital, with no fatal result. The mortality was thus 2.15 per cent. for the hospital first named; and 1.71 per cent. for both institutions. One death was due to exhaustion and two to perforation of intestines. No discrimination was made as to the physical character or class of the patients, nor as to the severity of the disease. Ninety-eight per cent. were characteristically prostrated by the malady, while but one was of the ambulatory type. The majority, in consequence of previous unfavorable surroundings, habits, or dyscrasiæ, had less than the average recuperative power.

Males preponderated, as only such are taken at the Alexian Hospital. It thus happens that one hundred and sixty were men, and only fifteen were women. The ages ranged from 14 to 50 years, the average being 25 years.

Prodromata in cases of this character are usually impossible to ascertain. The utter ignorance which is so often responsible for this malady and the carelessness which it involves renders patients and friends alike poor observers of the symptoms of the disorder. Questioning seldoms elicits anything satisfactory concerning the occurrence of epistaxis or even headache before an arrival at hospital; and reliance is only to be placed upon observations made after the admission of patients. Hence the phenomena of the initial stage may be disregarded, as statements made upon this head are untrustworthy.

Nor is it always practicable to ascertain the subjective conditions of the patients when stupor is a complication of their trouble. As near as could be determined abdominal tenderness was present in 15 cases; moderate tympanites in 29; tenderness with or without gurgling in right iliac region in 78; and troublesome sordes in 19. Epistaxis occurred in 40; vomiting in 14; diarrhœa in 95; constipation in 34; the remainder being irregular as to bowel movement.

The tongue coating varied as it usually does in the disorder, the frequent application of a mouth wash largely preventing the typical dryness and morbid accumulations upon the mucous surfaces. The maximum temperature in the cases that proceeded to convalescence was 105.5°; the minimum sub-normal being 96.5°; average stay in hospital thirty days.

In nine out of eleven relapses, the cause was directly traceable to disregard of the physician's orders concerning food or exercise. Usually indiscreet friends endanger the lives of the patients by smuggling indigestible edibles into the wards.

Careful notes were not made as to the typhoid eruption, but it was noticeable only in a very few cases. Splenic enlargement was frequent but variable.

The complications induced by the fever were as follows: complete aphonia 1; lobar pneumonia 3; lobular pneumonia 1; pleurisy 1; bronchitis, light cases, 3; vesical tenesmus 3; peritonitis 2; cervico-brachial neuralgia 1; hæmoptysis 1; hæmaturia 1; intercostal neuralgia 1; intestinal hæmorrhage 1; gastralgia 1; bed-sores 3.

The accompaniments of the typhoid fever were in some cases, intermittent fever 6, intermittent fever during convalescence 1, plumbism 2, arthritis 1, frontal head-injury 1, occipital head injury 2, hemicrania 1, alveolar abscess 1, acute conjunctivitis 1, spinal congestion 1, abscess of body 3, of larynx 1, of thigh 1, inguinal region 2.

The sequelæ were phlebitis 4, otitis media 2, tibial periostitis 1, parotiditis 2, suppurative phlebitis 1, suppurative periostitis of tibia 1, hemiplegia 1.

Delirium was marked in twenty cases; restlessness in about one-third; while profound stupor occurred in but one. The mental disquietude bore no relation whatever to the pyrexia.

Antipyrin was used in place of the older methods of temperature, such as the cold-pack, salicylates and quinine. This newly-discovered and valuable agent was resorted to usually when a persistent rise toward 103° was noted, or when the discomfort of the heat, the exhaustion it induced, or other indication justified its exhibition. Copious diaphoresis usually followed its administration, and in several cases the eruption peculiar to the medicine followed. This eruption was noticed as occurring more often in those whose skin was softened and tender from in-door life. Laborers did not suffer in this way as much. In two cases this skin trouble began at the groin, extending upward over the abdomen and chest. As a rule the chest and arms were the locations of the outbreak. The dose usually given was fifteen grains, smaller doses of twelve and ten grains, at intervals of two hours or thereabouts, being given if necessary. The first dose, as a rule, was all that was needed to lower the temperature from two to four degrees, three degrees being an average.

A tolerance of the drug when ingested was associated with a greater reduction in temperature from the same dose. In one case the sweating was so exhausting as to prevent the further use of antipyrin, but the diaphoresis induced varies within wide limits and such idiosyncrasy should be regarded where it exists.

It is, of course, an undetermined matter as to just what dangers attend high temperature *per se*. It is not to be expected that it is the only element needing control in the patient's behalf, but, so far as demonstrated by experimentation, the suppression of the pyrexia largely benefits the progress of the disorder. Antipyrin has, moreover, none of the disadvantages of the antipyretics formerly used. It is not irritating as are the salicylates ; it does not congest the brain as does quinine, nor has the fatal supervention of exhaustion been recorded against it as against the injudicious use of the cold-pack or bath. It would be well, however, to observe the condition of the thoracic viscera, as any depressant may induce collapse. Beaumetz and others call attention to the styptic properties of antipyrin; and the claim is made that intestinal hæmorrhage is controlled more quickly through its use than by any other means. Stimulants are antidotes to the undue effects of the drug.

The frequency of the pulse was reduced while its force was not affected when antipyrin was given therapeutically, and the diarrhœa was noticeably checked. Cephalalgia observed in the course of high temperature, was also relieved.

The medicine exerts no permanent effect upon the temperature range, for the afternoon rise will occur the next day after treatment just as though nothing had been given, but this does not lessen its value in enabling us to control immediate conditions.

Quinine was given only in tonic doses and usually during convalescence. Stimulants were used to bridge over the period of waning strength of the patient. One case was brought to the Alexian Hospital in the fifth week of typhoid fever, and the patient was found to be suffering from profound cinchonism. As many as 30 grains of quinine had been persistently taken. Discontinuance of the quinine was all that was needed to restore him to health.

The temperature record of a female patient three days after entering the Michael Reese Hospital was as follows:

| | | | | |
|---|---|---|---|---|
| 8.30 A. M. | 103° | 15 grains antipyrin. | | |
| 9.30 A. M. | 102.5° | 10 | " | " |
| 10.30 A. M | 101° | 10 | " | " |
| 11.30 A. M. | 100° | | | |
| 5.00 P. M. | 103° | 15 grains antipyrin. | | |
| 6.00 P.M. | 101.4° | 10 | " | " |
| 7.00 P. M | 99.2° | | | |

In only one instance was the medicine rejected by the stomach.

A chart is herewith given of the daily range of temperature in a male case at the Alexian Hospital, the dotted lines indicating the reduction of temperature induced when the new remedy was used.

## Figures and Tables

**Figure f1:**